# Differential Calcium Signaling by Cone Specific Guanylate Cyclase-Activating Proteins from the Zebrafish Retina

**DOI:** 10.1371/journal.pone.0023117

**Published:** 2011-08-02

**Authors:** Alexander Scholten, Karl-Wilhelm Koch

**Affiliations:** 1 Institute of Biology and Environmental Science, Carl von Ossietzky University Oldenburg, Oldenburg, Germany; 2 Research Center Neurosensory Science, Carl von Ossietzky University Oldenburg, Oldenburg, Germany; 3 Center of Interface Science, Carl von Ossietzky University Oldenburg, Oldenburg, Germany; Dalhousie University Canada

## Abstract

Zebrafish express in their retina a higher number of guanylate cyclase-activating proteins (zGCAPs) than mammalians pointing to more complex guanylate cyclase signaling systems. All six zGCAP isoforms show distinct and partial overlapping expression profiles in rods and cones. We determined critical Ca^2+^-dependent parameters of their functional properties using purified zGCAPs after heterologous expression in *E.coli*. Isoforms 1–4 were strong, 5 and 7 were weak activators of membrane bound guanylate cyclase. They further displayed different Ca^2+^-sensitivities of guanylate cyclase activation, which is half maximal either at a free Ca^2+^ around 30 nM (zGCAP1, 2 and 3) or around 400 nM (zGCAP4, 5 and 7). Zebrafish GCAP isoforms showed also differences in their Ca^2+^/Mg^2+^-dependent conformational changes and in the Ca^2+^-dependent monomer-dimer equilibrium. Direct Ca^2+^-binding revealed that all zGCAPs bound at least three Ca^2+^. The corresponding apparent affinity constants reflect binding of Ca^2+^ with high (≤100 nM), medium (0.1–5 µM) and/or low (≥5 µM) affinity, but were unique for each zGCAP isoform. Our data indicate a Ca^2+^-sensor system in zebrafish rod and cone cells supporting a Ca^2+^-relay model of differential zGCAP operation in these cells.

## Introduction

Neuronal calcium sensor proteins regulate different neuronal functions by interactions with specific targets [1], [2]. They can be classified in five subgroups having members in yeast, nematodes, insects, fishes and mammals. Two subgroups, the recoverins and the guanylate cyclase-activating proteins (GCAPs), are primarily expressed in rod and cone photoreceptor cells of the vertebrate retina, where they control enzyme activities of rhodopsin kinase and guanylate cyclases by sensing changes of intracellular Ca^2+^ during illumination. Light causes a drop of intracellular Ca^2+^, which triggers activation of membrane bound guanylate cyclases (GC) in rod and cone cells. This effect is mediated by GCAPs and has mainly been studied with the mammalian isoforms GCAP1 and GCAP2 [3]–[6].

A larger variety (six to eight) of GCAP isoforms was found in pufferfish (*Fugu rubripes*) [7], carp (*Cyprinus carpio*) [8] and zebrafish (*Danio rerio*) [7]–[9] and mRNA transcripts have been localized in photoreceptor cells of the adult fish retina, whereby the four isoforms zGCAP3, 4, 5 and 7 exhibit a cone specific expression pattern. In situ hybridization studies showed that zGCAP3 and zGCAP5 are dominantly present in all four cone cell types, red- and green-sensitive double cones, blue-sensitive long single cones and UV-sensitive short single cones [[10], (Rätscho, N., Scholten, A. and Koch, K.-W., unpublished observation)]. Transcription of zGCAP4 was most intense in double cones and long single cones, significantly less in short single cones, whereas transcripts of zGCAP7 were weakly stained in all cone cell types [7]. A different transcription pattern was observed for zGCAP1 and 2 showing expression in rods and short single cones [[7], [11], (Rätscho, N., Scholten, A. and Koch, K.-W., unpublished observation)]. A recent study on zebrafish larvae further showed that zGCAP3, zGCAP4 and zGCAP7 were expressed already 3–4 days post fertilization (dpf) and the expression onset overlapped with the temporal transcription start of three membrane bound sensory guanylate cyclases zGC1–3 [9].

An initial biochemical characterization of zGCAP3 and zGCAP4 revealed that both proteins undergo Ca^2+^-induced conformational changes and thereby activate membrane bound guanylate cyclases at low [Ca^2+^] [10], [11]. A recent study that combined biochemical and electrophysiological methods explored the GCAP/GC-mediated cGMP synthesis in rod and cones from the carp retina showing a 5–10 times higher cGMP synthesis rate in cones than in rods [8].

Unresolved issues of cone phototransduction include the molecular details enabling cones to operate over a much larger light intensity range than rods and the nonsaturating behaviour of the cone light response [12].

In mammalian photoreceptors regulation of light sensitivity is to a significant part mediated by changes in cytoplasmic [Ca^2+^] [13] and includes also translocation processes of signaling proteins [14]. Ca^2+^-sensor proteins in rod and cone cells detect changes in cytoplasmic [Ca^2+^] and regulate their target proteins in a Ca^2+^-dependent manner [1]–[6]. The seemingly redundant expression of GCAP isoforms in vertebrate rod and cone cells has led to several biochemical and physiological studies that demonstrated distinct roles of GCAP1 and GCAP2. A characteristic feature of the GCAP1-GCAP2-tandem is their difference in Ca^2+^-sensitive regulation of GCs [15]–[17], which apparently is the molecular basis of their different effects in shaping the light response of photoreceptor cells [18]–[21]. These findings collectively support a “Ca^2+^-relay model” of GCAP action, where a fall in intracellular [Ca^2+^] triggers the sequential activation of GCAP1 and GCAP2 [22], [23].

The large variety of zGCAP isoforms in zebrafish cone cells raises the question, whether cone specific isoforms of zGCAPs differ in their Ca^2+^-sensitive properties enabling also a step by step activation of guanylate cyclases in a sequential order. We addressed this question by investigating the Ca^2+^-sensitive properties of all zGCAPs in a comparative study.

## Materials and Methods

### Cloning and site-directed mutagenesis

The cloning of zGCAP4 has been described elsewhere [11]. The coding sequences of zGCAP1, 2, 3, 5 and 7 were amplified from a zebrafish retinal cDNA library (number 760, Deutsches Ressourcenzentrum für Genomforschung GmbH, Berlin; used for zGCAP3 and 5) or from cDNA first strand preparations of adult retina (zGCAP1, 2 and 7) by polymerase chain reaction employing the following primers 5′-AAACATATGGGCAATTCAACGGGCAGC-3′ and 5′-AACGAATTCTTAAACGCTGTGTCTCCGGTTATG-3′ (zGCAP1), 5′-TTCCATATGGGTCAGCGGCTCAGCG-3′ and 5′-GTGGAATTCAAAAGTTGGCGCTGCGGC-3′ (zGCAP2), 5′-GGACATATGGGCGCCCACGCAT-3′ and 5′-GTTCCCGTCCACGAATTCACTCCTTC-3′ (zGCAP3), 5′-CTCCATATGGGGGACTCCTCCAGCAT-3′ and 5′-AGTGAATTCATGCTTGATCCTCGATGATC-3′ (zGCAP5), 5′-AAACATATGGGCCAGAATCAAAGCGATG-3′ and 5′-AAAGAATTCATGTTTTCTTCCCCAAGTTGT-3′ (zGCAP7). For heterologous expression in *E.coli* the amplified PCR product was ligated into a pET-expression vector using *NdeI* and *EcoRI* restriction sites. Plasmid preparation, restriction analysis and transformation were done according to standard protocols. Sequences of all zGCAP forms were verified by DNA sequencing.

### Heterologous expression and purification of zGCAP forms

Large amounts of non-myristoylated zGCAPs were obtained by overexpression in BL21 *E.coli* cells. Expression was started at an OD_600_ of 0.6 by induction with IPTG and bacteria were grown for 4 hours at 37°C. Bacteria were harvested by centrifugation and stored until use at −80°C. After thawing and cell lysis zGCAP forms were isolated from the soluble cell fraction (zGCAP3 and 4) or from inclusion bodies (zGCAP1, 2, 5 and 7). Purification was a combination of anion exchange chromatography on a HiLoad 26/10 Q sepharose-column and of size exclusion chromatography on a HiLoad 26/60 Superdex 75-column (both from Amersham). The columns were equilibrated in 20 mM Tris-HCl pH 7.5, 1 mM DTT and NaCl in different concentrations. Before the samples were applied to size exclusion chromatography proteins were precipitated by ammonium sulfate to reduce volume. Prior to chromatography steps insoluble proteins from inclusion bodies were completely denatured using 6 M guanidine hydrochloride and refolded during dialysis against chromatography buffer (20 mM Tris-HCl, pH 7.5, 150 mM NaCl, 1 mM DTT) including one buffer change.

### Guanylate cyclase assay

Guanylate cyclase activity in bovine rod outer segment (ROS) membranes originates mainly (ca. 96%) from ROS-GC1 and to a lesser extent (4%) from ROS-GC2 [24]. We used washed bovine ROS membranes that were stripped of endogeneous GCAPs to measure GC activities after reconstitution with purified zGCAPs according to a detailed protocol that was published before [25] with the following modifications: for the determination of EC_50_-values the zGCAP concentration was varied between 0.25 and 10 µM. Ca^2+^-dependent GC activities were obtained by keeping zGCAP concentrations fixed at 10 µM and varying the free [Ca^2+^] using a K_2_H_2_EGTA/CaH_2_EGTA buffer system as described previously [11], [24]. The free Mg^2+^-concentration was 1 mM. All incubation steps were performed under very dim red light, the reactions were stopped and further processed for analysis as described.

### Limited proteolysis

GCAP samples were treated with trypsin similarly to [26]. We used a ratio of 300∶1 (GCAP: trypsin) in a volume of 50 µl and added either CaCl_2_ or EGTA to a final concentration of 1 mM. The reaction was stopped after 2, 5 or 10 minutes by boiling and further analyzed by sodium dodecylsulfate (SDS) polyacrylamide gel electrophoresis.

### High performance liquid chromatography

Purified protein samples (20 µg) were analyzed for the presence of monomeric and/or dimeric zGCAP forms by employing size exclusion chromatography on a BioSep-SEC S2000 column (Phenomenex, Aschaffenburg, Germany) including molecular mass standards as described [26].

### Tryptophan (Trp) fluorescence spectroscopy

Intrinsic Trp fluorescence measurements were performed with a spectrometer from photon Technology International (pTI) essentially as described before [25], [26]. Data recording and analysis was done with the pTI software package FELIX32. Lyophilized samples of zGCAPs were dissolved in bidistilled water containing 1 mM DTT at a concentration of 70–120 µM. Shortly before recording the spectrum the protein stock was diluted in fluorescence buffer (80 mM Hepes pH 7.5, 40 mM KCl, 1 mM MgCl_2_, 1 mM DTT) at a final concentration of 0.7–1.2 µM. The excitation wavelength was set to 280 nm, the emission spectrum was recorded from 300–420 nm. Different free [Ca^2+^] were adjusted by a set of Ca^2+^/EGTA buffer solution (see above).

### Determination of Ca^2+^-binding constants

Ca^2+^-binding to zGCAPs was monitored using a previously described chelator assay [27]–[29]. The method is based on the competition for Ca^2+^ between the protein and a chromophoric chelator whose absorbance changes upon Ca^2+^ binding. Similar amounts (10–50 µM) of the chelator 5,5′Br_2_-BAPTA and protein are titrated with Ca^2+^ and the binding of the cation to the chelator is monitored by UV absorbance at 263 nm. Data were fitted by employing a Newton-Raphson direct least-square fitting procedure implemented in the CaLigator software [27]. Details of the titration procedure and evaluation of the data obtained with zGCAPs was performed as described recently for mammalian GCAP1 mutants [30]. In addition, after obtaining the macroscopic binding constants (K_i_, i = 1,2,…etc.) for each binding site as described [30], these values were used to assess the degree of saturation for each zGCAP variant as a function of free [Ca^2+^], hence yielding the apparent affinity constants. For the fitting the precise concentration of each protein sample stock was determined by amino acid analysis after acid hydrolysis (Biomedical Center, Uppsala, Sweden).

### Other methods

Standard biochemical techniques as SDS polyacrylamide gel electrophoresis and determination of protein content were done according to well established protocols in our laboratory [5], [15], [16], [24]–[26]. For protein determination of purified zGCAP samples we measured light extinction at 280 nm using isoform specific extinction coefficents evaluated in our laboratory. Heterologous expression, purification and use of bovine GCAP1 and GCAP2 (bGCAP) samples were exactly as previously described [15], [16], [24]–[26].

## Results

### Heterologous expression and purification of zGCAPs

**Figure 1:**
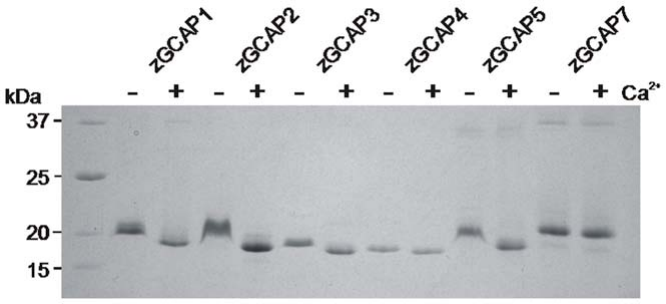
Electrophoretic analysis of purified zGCAPs. Two µg of purifed nonmyristoylated zGCAPs were applied on a SDS polyacrylamide gel (15% acrylamide) in the absence (−, 1 mM EGTA) and presence (+, 1 mM CaCl_2_) of Ca^2+^. EGTA-samples also contained 1 mM MgCl_2_. After electrophoresis the gel was stained with Coomassie Blue.

All six zebrafish specific GCAP forms could be purified from bacterial lysates using anion exchange chromatography, ammonium sulfate precipitation and size exclusion chromatography. Although all isoforms could be purified from soluble cell fractions higher yields were obtained by purifying and renaturing zGCAP1, 2, 5 and 7 from inclusion bodies. In control experiments no differences were observed between proteins from soluble fractions or from inclusion bodies. All Ca^2+^-loaded zGCAPs have an apparent molecular mass of 19–21 kDa as analysed by SDS polyacrylamide gel electrophoresis and the electrophoretic mobility became less in the absence of Ca^2+^ by adding EGTA. Interestingly, this Ca^2+^-shift differed among the tested zGCAPs as it was most prominent for zGCAP2 and zGCAP5 (Figure 1 and Table 1). Furthermore, we observed that adding Mg^2+^ to the sample buffer containing EGTA enhanced the Ca^2+^-shift in particular for zGCAP2 and zGCAP5 (as shown in Figure 1), which points to the Mg^2+^-binding properties of GCAPs as described previously for mammalian GCAPs [17]. We used non-myristoylated zGCAPs in the present study for the following reasons: (a) not all zGCAPs contain an ideal consensus site for myristoylation and so far it is unknown, whether zGCAPs expressed in zebrafish retina are myristoylated or not; (b) when coexpressed with a myristoyltransferase in *E.coli*, we were not able to confirm myristoylation for all zGCAPs, not even after creating a consensus site for myristoylation by site-directed mutagenesis (unpublished observation). So far, only for zGCAP4 we were able to investigate the myristoylated and non-myristoylated forms of the protein in more detail and found no significant differences in key properties of its Ca^2+^-sensor function [11]. However, we cannot exclude that some zGCAP isoforms exhibit different properties, when expressed as myristoylated variants in zebrafish cones.

**Table 1:** Comparison of biochemical properties of bovine (b) and zebrafish (z) GCAPs.

properties	bGCAP1^1^	bGCAP2^2^	zGCAP1	zGCAP2	zGCAP3	zGCAP4	zGCAP5	zGCAP7
“Ca^2+^-shift” (ÄkDa)	4–5	4–5	2	3	<1	1	2	<1
Ca^2+^-bound state more stable against proteolysis by trypsin	+	+	+	+	+	+	+	+
change in Trp fluorescence <1 µM [Ca^2+^]_f_	drop	drop	increase	increase	increase	increase	no change	increase
change in Trp fluorescence >1 µM [Ca^2+^]_f_	increase	no change	increase	increase	increase	increase	increase	no change
effect of Mg^2+^ on Trp fluorescence	+	+	−	−	−	−	−	+
Ca^2+^-dependent changes in monomer/dimer-ratio	−	+	+	−	−	−	−	+

1 and

2 data taken form references 3, 16, 26, 32 and 34.

### Activation of membrane bound guanylate cyclase by zGCAPs

**Figure 2:**
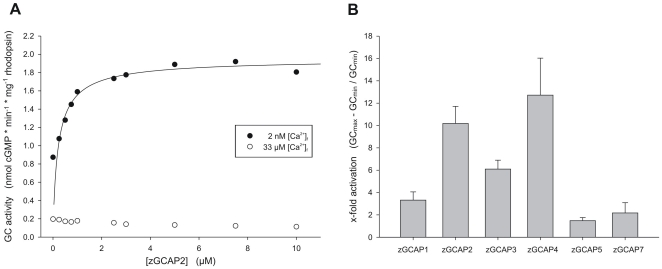
Guanylate cyclase activation by zGCAPs. Purified samples of zGCAPs were incubated with washed ROS membranes from bovine rod outer segments. The prominent GC in these membranes is ROS-GC1 (GC-E) [24]. A. In order to determine EC_50_ values bovine ROS-GC1 in native membranes was reconstituted with increasing concentrations of zGCAPs at low free [Ca^2+^] of 2 nM. A representative plot is shown for zGCAP2 with data obtained at low (black circles) and high (33 µM) [Ca^2+^] (open circles). B. GC activities were measured after reconstitution with 10 µM of each zGCAP isoform at high and low free [Ca^2+^], 33 µM and 2 nM, respectively. Activities are expressed in x-fold activation for each zGCAP (GCmax−GCmin divided by GCmin). Data are the mean of 3–5 sets ± s.d.

Regulatory properties of zGCAPs were tested using washed bovine ROS membranes as a source for GCs. Tests were run in different ways leading to three parameters: the EC_50_ value gives the particular zGCAP concentration that half-maximally activates GCs, the IC_50_ value gives the [Ca^2+^] at which the GC activity is halfmaximal and the x-fold activation is expressed as GC_max_−GC_min_ divided by GC_min_ (Table 2). EC_50_ values of zGCAPs were in the range between 0.2 and 3 µM (Table 2 and Figure 2A for a representative example) and were comparable to the EC_50_ of bovine GCAP1 and GCAP2, 0.9 and 0.2 µM, respectively [31]. Significant differences however were observed in the IC_50_ values and the x-fold activation (Table 2). The strongest activators were zGCAP2 and zGCAP4 with 10- to 13-fold activation (Figure 2B). Less strong, but similar to the bovine ortholog was zGCAP1. A slightly stronger activator was zGCAP3, but rather weak activation of two-fold or less was observed with zGCAP7 and zGCAP5.

**Table 2:** GC activating properties of GCAPs obtained with washed bovine ROS membranes.

properties	bGCAP1	bGCAP2	zGCAP1	zGCAP2	zGCAP3	zGCAP4	zGCAP5	zGCAP7
EC_50_-value (µM GCAP)	0.9^1^	0.2^1^	3.0	0.2	0.7	0.7^3^	n.d.	1.2
IC_50_-value (nM [Ca^2+^]_f_)	1,100^2^	215^2^	30	35	25	520^3^	440	180
x-fold activation^4^	3-fold^2^	9-fold^2^	3-fold	10-fold	6-fold	13-fold^3^	1.5-fold	2-fold

1 data taken from ref. 31.

2 data taken from ref. 16.

3 data taken from ref. 11.

4 x-fold activation is expressed as GC_max_−GC_min_/GC_min_; GC_max_: GC activity at maximum; GC_min_: GC activity at minimum.

### Ca^2+^-sensitive regulation of membrane bound guanylate cyclase by zGCAPs

**Figure 3:**
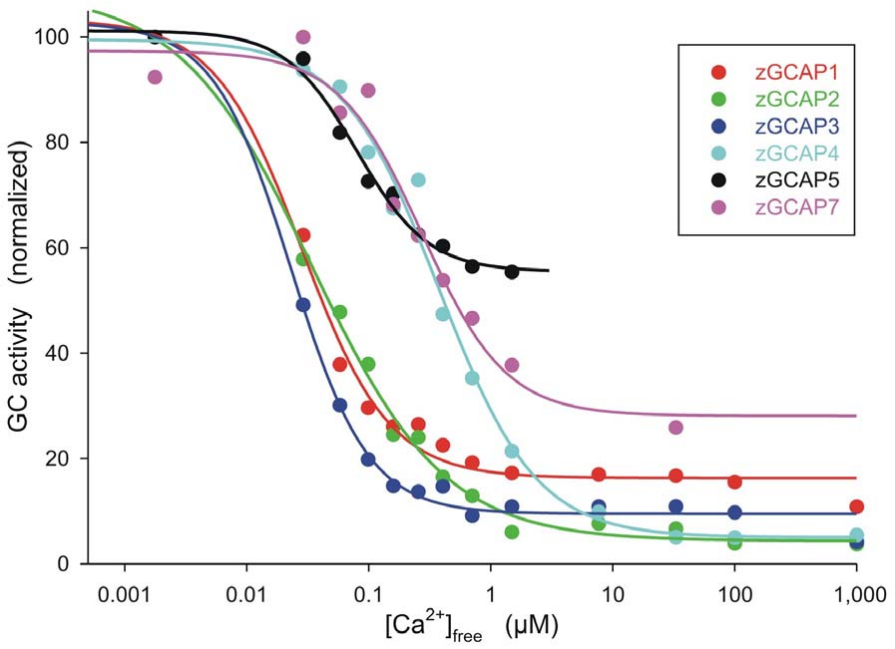
Ca^2+^-sensitive regulation of GC activity by zGCAPs. Ca^2+^-dependent activation profile of native bovine ROS-GC1 by different zGCAP isoforms (10 µM); free [Ca^2+^] was adjusted by a Ca^2+^/EGTA buffer system and the maximal activation was normalized to 100%. The IC_50_ value of each zGCAP is listed in Table 2. Data are representative of at least three independent replicates.

A characteristic feature of mammalian GCAPs is their Ca^2+^-sensitive regulation of membrane bound GC activity. The IC_50_ values of zGCAPs could be separated in two groups; one group consisting of zGCAP1, 2, and 3 had IC_50_ values around 30 nM free [Ca^2+^], the second group of zGCAP4, 5 and 7 had IC_50_ values between 180 and 520 nM centered around 400 nM (Table 2 and Figure 3). Taking into account the different expression profiles of zGCAPs [9], [10], every cone cell type seems to express at least one pair of zGCAPs with different Ca^2+^sensitivities (see also Discussion below).

### Ca^2+^-induced conformational changes in zGCAPs

Ca^2+^-induced conformational changes of GCAPs are thought to trigger their biological function [3]–[6], [11]. We performed a series of tests to investigate, how zGCAPs undergo Ca^2+^-dependent conformational changes. First, zGCAPs exhibited a Ca^2+^-dependent electrophoretic mobility shift as shown in Figure 1. Second, limited proteolysis with trypsin allows detecting differences in protease accessibility, when Ca^2+^ is present or absent [25], [32]. All Ca^2+^-bound zGCAPs were more stable against proteolysis than their Ca^2+^-free counterparts (Table 1). These results indicated that changing [Ca^2+^] lead to a change in protein conformation in all zGCAPs.

**Figure 4:**
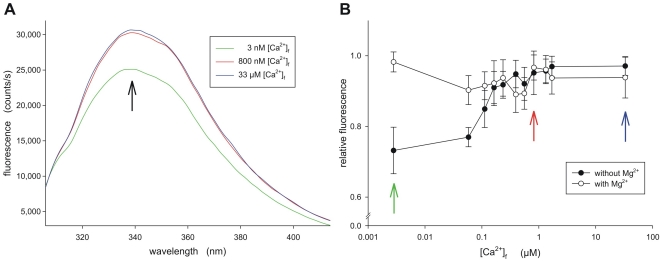
Trp fluorescence emission of zGCAP7. A. Exemplary fluorescence emission scans of zGCAP7 at three different free [Ca2+], 3 nM, 800 nM and 33 µM. No Mg^2+^ was present. B. The relative fluorescence at 336 nm of zGCAP7 (approx. 1 µM) was measured at different free [Ca^2+^] in the presence and absence of 1 mM Mg^2+^ as indicated. Coloured arrows indicate the data of corresponding scans in panel A. Values were normalized to the highest obtained value (see black arrow in panel A) of each data set. Each data point is the mean of four separate determinations ± s.d.

Third, Ca^2+^-induced conformational changes in mammalian GCAP1 and GCAP2 can be monitored by intrinsic Trp fluorescence allowing the determination of the free [Ca^2+^] that triggers the change [3], [26], [33], [34]. We recorded fluorescence emission between 300 and 420 nm for all zGCAPs. Representative fluorescence emission scans of zGCAP7 are shown in Figure 4A. The relative fluorescence intensity was at low free [Ca^2+^] (2–3 nM) for nearly all zGCAPs at a minimum. It constantly changed to higher intensity values at increasing [Ca^2+^] (Table 1). An exception was observed with zGCAP5 that showed no change in fluorescence intensity below 1 µM [Ca^2+^] and with zGCAP7 that showed no change above 1 µM [Ca^2+^] (Table 1). For comparison we included data from the literature that were obtained with bovine GCAP1 and 2. In this aspect zGCAP7 was similar to bovine GCAP2, whereas zGCAP5 was unique. Changes in Trp fluorescence below 1 µM indicated that zGCAPs undergo conformational changes in a physiologically relevant range of the free [Ca^2+^], which is consistent with the IC_50_ values reported above.

Recent work by Peshenko and Dizhoor [34], [35] has demonstrated that GCAPs can bind Mg^2+^ in exchange for Ca^2+^, when [Ca^2+^] is kept low. By this action Mg^2+^ can affect the intrinsic Trp fluorescence. For instance, it abolishes the Ca^2+^-dependent changes in fluorescence intensity denoted as phase I [17]. This effect was prominently visible with bovine GCAP1 and GCAP2 as previously reported, but we did not see it with zGCAPs. Only the Trp fluorescence of zGCAP7 was sensitive to Mg^2+^, which is shown in Figure 4. Below 1 µM free [Ca^2+^] the relative Trp fluorescence emission of zGCAP7 is lower without than with Mg^2+^.

### Ca^2+^-dependent monomer-dimer formation of zGCAPs

**Figure 5:**
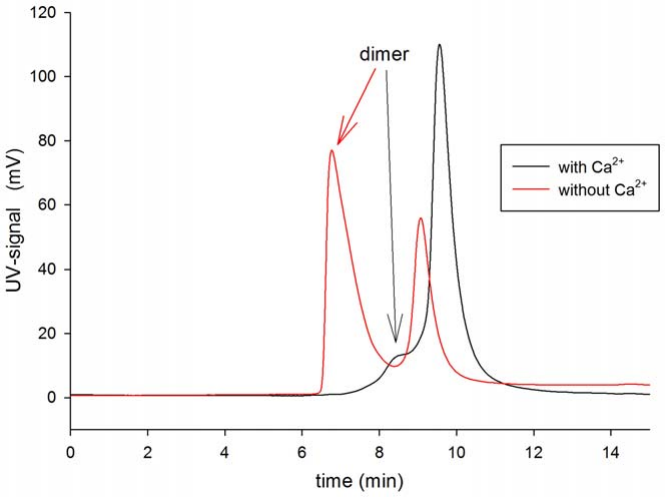
Analytical size exclusion chromatography of zGCAP7. Samples of zGCAP7 were loaded on a BioSep-SEC S2000 column and the monomer-dimer equilibrium was analyzed by performing the runs either in the presence of 0.5 mM CaCl_2_ or 0.5 mM EGTA (red trace). Elution of proteins was detected at 280 nm (monitored as a mV-signal). Elution of a zGCAP7 dimer is indicated by an arrow.

Changing [Ca^2+^] influences the monomer-dimer equilibrium of bovine GCAP1 and GCAP2 in different ways [26], [36]. We therefore analyzed the monomer-dimer equilibrium of zGCAPs by size exclusion chromatography showing that only zGCAP1 and zGCAP7 dimerize in a Ca^2+^-dependent manner similar as it was observed with bovine GCAP2 (Table 1). An exemplary size exclusion chromatography of zGCAP7 is presented in Figure 5 showing the elution of Ca^2+^-bound zGCAP7 at 9.5 min corresponding to a zGCAP7 monomer. The small shoulder at 8.5 min resulted probably from a minor fraction of dimeric zGCAP7. Complexing Ca^2+^ by addition of EGTA led to a chromatographic resolution of two peaks with retention times at 6.8 min and 9 min, which would correspond to dimeric and monomeric zGCAP7, respectively. The slight shift in retention times of monomer and dimer forms, when the [Ca^2+^] was changed (for example from 9.5 to 9 min) mirrors the Ca^2+^-induced conformational change in zGCAP7 leading to a different hydrodynamic volume of the protein. Other zGCAP forms did not undergo a monomer-dimer equilibrium shift; instead they remained to a large extent in a monomeric state as this was seen with zGCAP7 in the presence of Ca^2+^ (Figure 5, black trace). Only zGCAP1 formed to some extent dimers (about 18%).

### Ca^2+^-binding of zGCAPs

**Figure 6:**
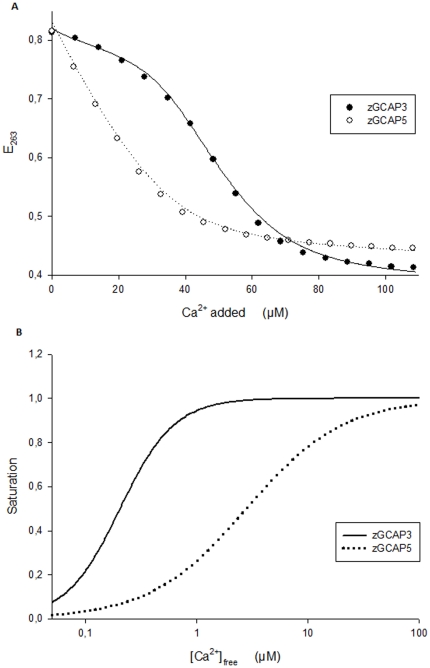
Ca^2+^-binding to zGCAPs. A. Ca^2+^-titration curves for zGCAP3 and zGCAP5. Experimental points obtained by the chelator assay are shown as indicated and were fitted as described previously [27]–[30]. Protein concentration was 14.4 µM for zGCAP3 and 11.7 µM for zGCAP5. B. Ca^2+^ saturation curves of zGCAP3 and zGCAP5 corresponding to the titrations shown in A. Determination of the point of halfmaximal saturation yielded the apparent K_D_ values listed in Table 4.

Direct binding of Ca^2+^ to zGCAPs was tested by the chelator assay measuring the competition of zGCAPs and the chromophoric chelator 5,5′Br_2_-BAPTA for Ca^2+^ [27]–[29]. Two examples of titration curves obtained with zGCAP3 and zGCAP5 are shown in Figure 6A yielding apparent macroscopic affinity constants by computer fitting [27] that are listed in Table 3. The Ca^2+^-titration curves could be best fitted by a model assuming three Ca^2+^-binding sites, except for zGCAP7 for which four Ca^2+^-binding sites gave the best fit result. All zGCAPs except zGCAP5 and zGCAP2 displayed one high affinity site with an apparent affinity constant K^h^≤100 nM. At least one constant of medium affinity (K^m^ = 0.1–5 µM) was obtained for all zGCAPs and one low affinity site was measured with zGCAP1, zGCAP2 and zGCAP5 (K^l^≥5 µM). A particular case was observed with zGCAP7 that contained one high affinity and three medium affinity sites (Table 3). The results obtained with zGCAP4 allowed only giving a range of macroscopic binding constants that broadly fit into the categories mentioned above (see footnote to Table 3). Transforming the data of the chelator titration assay into Ca^2+^-saturation curves (Figure 6B) allowed us to determine a macroscopic affinity constant as an apparent K_D_ for the binding of Ca^2+^ to zGCAPs (Table 4). Among different zGCAP isoforms these values span a range from 70 nM to 3.9 µM Ca^2+^. Interestingly, the apparent Ca^2+^-affinities did not match with the IC_50_ values of GC regulation (compare with Table 3). Instead they were in all cases much lower and the apparent K_D_s differed from the IC_50_ value by a factor of 2.3 (zGCAP1) up to a factor of 48 (zGCAP2).

**Table 3:** Summary of results from the chelator assay determining Ca^2+^-binding affinities including macroscopic binding constants.

Isoform	logk1	logk2	logk3	logk4
zGCAP1	7.15±0.18	1.37±0.60	5.77±1.39	
zGCAP2	5.33±0.11	6.02±0.12	4.31±0.25	
zGCAP3	6.06±0.29	7.25±0.14	5.52±0.23	
zGCAP4	see below^1^	see below^1^	see below^1^	
zGCAP5	5.42±0.12	1.49±0.75	6.53±0.49	
zGCAP7	6.41±2.13	6.44±0.27	7.75±1.12	6.24±1.77

1 Macroscopic binding constants were measured from a total set of 14 determinations and exhibited large scattering in the following ranges: logk1 = ≥8; logk2 = 5–7; logk3 = 2–6.

**Table 4:** Summary of macroscopic apparent K_D_ values obtained from Ca^2+^-saturation curves.

Isoform	k_D_ [µM]
zGCAP1	0.07±0.03
zGCAP2	1.67±0.13
zGCAP3	0.19±0.05
zGCAP4	n.d.
zGCAP5	3.87±1.0
zGCAP7	1.71±0.34

## Discussion

The central conclusion of the present work is that the variety of GCAPs in the zebrafish retina reflects a Ca^2+^-sensor system with different biochemical properties. Most significant are differences in the IC_50_ values (Table 2 and Figure 3) that cluster at rather distant free [Ca^2+^] but fit into the physiological range of [Ca^2+^] in a vertebrate photoreceptor cell.

Direct measurements of cytoplasmic [Ca^2+^] in zebrafish cones (and rods) are still missing, but Ciluffo et al. suggested from their measurements on visible cones a mean dark value around 400 nM [37]. They further observed that sufficiently high background light can cause a transient increase of [Ca^2+^] which is followed by the well understood light-triggered *decrease* of cytoplasmic [Ca^2+^] in photoreceptor cells via the operation of the Na^+^/Ca^2+^, K^+^-exchanger. This complex pattern of changes in intracellular [Ca^2+^] had previously been detected in cones from the zebrafish mutant *nof* [38] and, more recently, had been described in UV-sensitive cones [39]. All zGCAPs underwent Ca^2+^-induced conformational changes and regulated membrane bound GC between 30 and 500 nM [Ca^2+^], but why do cone cells express more than one form of zGCAP?

**Figure 7:**
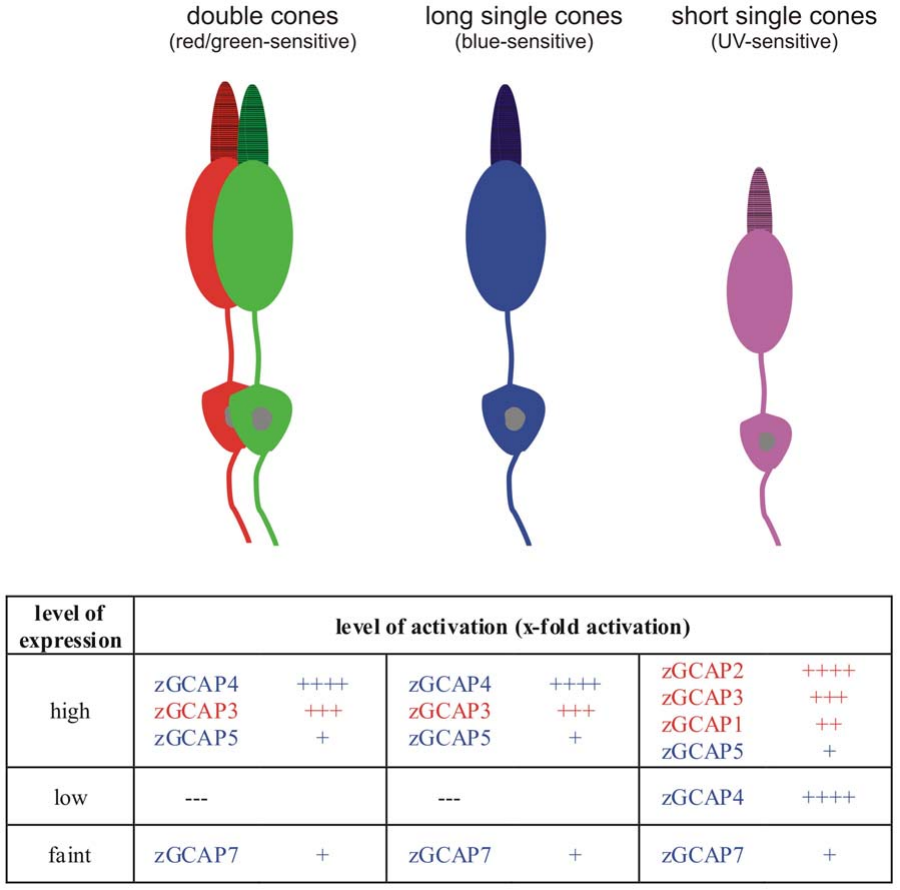
Schematic summary of zGCAP expression in different cone types. Double cones (long-wavelength sensitive), long single cones (short-wavelength sensitive) and short single cones (UV-sensitive) are drawn schematically in the upper part. The lower part compares the expression profiles of zGCAPs with their activation profiles. The indicated level of activation (x-fold) corresponds to >10-fold: ++++; 5–10-fold: +++; 3–5-fold: ++; <3-fold +. Isoforms of zGCAPs regulating GC with a low IC_50_ value (around 30 nM free [Ca^2+^]) are written in red, isoforms regulating GC with a high IC_50_ around 400 nM [Ca^2+^] are written in blue. Details are described and discussed in the main text.

Our data suggest that the different cone subtypes harbour combinations of zGCAPs that exhibit complementary properties. Figure 7 gives an overview of zGCAP expression and the level of activation that is achieved with the particular isoform. For example, strong transcription signals of zGCAP1, 2, 3 and 5 were observed in UV cones [7], [9], but significantly lower transcript levels were observed for zGCAP4 and 7 (Rätscho, N., Scholten, A. and Koch, K.-W., unpublished observation). Thus, UV cones would express one isoform (zGCAP2) with high and two (zGCAP1 and 3) with moderate x-fold activation (Table 2). The Ca^2+^-sensing and GC-activating properties further support this conclusion as the IC_50_ values of zGCAP1, 2 and 3 were similar and differed from those of zGCAP4, 5 and 7. The low IC_50_ values of UV-cone specific zGCAPs indicate further that the Ca^2+^-feedback on cGMP synthesis in order to operate efficiently would require a larger drop in cytoplasmic [Ca^2+^] than in other cone types, but so far a comparative analysis of changes in cytoplasmic [Ca^2+^] in different cone types is missing.

In long single cones and double cones transcription of zGCAP3, zGCAP4 and zGCAP5 [7], [9] appears strong, but that of zGCAP7 is weak. Again, we have a similar, but not identical pattern as in UV cones: one strong activator (zGCAP4), one moderate activator (zGCAP3) and one weak activator (zGCAP7) of GC are found in the same cell types. Further, the range of [Ca^2+^], where these zGCAPs dynamically regulate GC, is expanded with IC_50_ values between 30 and 520 nM.

Measurements of the apparent Ca^2+^-affinity of each zGCAP were broadly consistent with the Ca^2+^-sensitive regulation of GC, since the apparent binding constants were in the nanomolar to lower micromolar range (Table 4). One peculiar case was observed with zGCAP7, for which we obtained the best fitting result assuming four Ca^2+^-binding sites. We can only speculate on the interpretation of this result, since the first EF-hand is considered to be nonfunctional in all zGCAPs including zGCAP7 [7], although this has not experimentally proven and waits further investigation. However, one might argue that the Ca^2+^-free zGCAP7 is present as a dimer at the start of the Ca^2+^-titration and is shifted to the monomeric form at increasing [Ca^2+^]. We cannot exclude that the monomer-dimer equilibrium of zGCAP7 (Table 1 and Figure 5) interferes with the Ca^2+^-binding events during the titration, but in case of zGCAP1 that also formed dimers in the presence of EGTA we obtained a satisfying fit with three Ca^2+^-binding sites.

However, apparent Ca^2+^-affinities do not always match with the corresponding IC_50_ values. Therefore, it seems that the interaction of zGCAPs with the target GC changes the affinity of the Ca^2+^-sensor (i.e. zGCAP) for Ca^2+^ leading to a zGCAP/GC complex that has a higher Ca^2+^-affinity than the isolated protein. This form of intermolecular tuning of a Ca^2+^-sensor by its target has been previously observed with calmodulin and is an effective way to allow one small regulatory protein (calmodulin) to interact with a large variety of proteins in a cell [40]–[42]. The effect of mutually dependent Ca^2+^-affinities can be explained by free-energy coupling [43] meaning that binding of Ca^2+^ to calmodulin influences its affinity to a target in the same manner as the interaction of calmodulin with a target changes its affinity for Ca^2+^. The same behavior has been hypothesized for GCAP1/GC interaction [30], but it has been challenged in a recent study on transgenic mice showing that two different GC isoforms are regulated by the same mammalian GCAP with identical sensitivities [44]. The target dependent modulation of the Ca^2+^-sensitivity of zGCAPs might become even more complex, if we consider that the zebrafish retina expresses three sensory GCs, one being cone specific and two being expressed in rods and UV cones [9], [38], [45]. It awaits further analysis to test the combinations of six zGCAP and three GC forms.

Limited proteolysis in the presence and absence of Ca^2+^ and Trp fluorescence spectroscopy indicated that all zGCAPs exhibited a Ca^2+^-induced conformational change. Ca^2+^-dependent conformational changes belong to the key properties of Ca^2+^-sensors and are thought to represent the activating trigger step in a GCAP molecule that activates its associated target GC [3]–[5], [22]. Intrinsic Trp fluorescence monitors movements of amino acid side chains in proteins and was particularly useful for correlating conformational changes in mammalian GCAPs with changing [Ca^2+^] [26], [32]–[35]. Of particular interest was the observation that the pattern of Trp emission of most zGCAPs (not zGCAP7) did not change by the presence of Mg^2+^, which clearly differs from the observation made with bovine GCAP isoforms [34], [35]. For example, bovine GCAP1 relative fluorescence emission changes below 1 µM [Ca^2+^] (designated as phase I) correlate with amino acid side chain movements around Trp 21 and 51 and changes above 1 µM [Ca^2+^] (designated as phase II) with movements around Trp 94 [35]. All zGCAPs (with the exception of zGCAP7, see below) harbour a Trp around position 21, but they lack a Trp at position 51 that was shown to be sensitive to Mg^2+^ in bovine GCAP1 [34], [35]. These facts provide an explanation why we did not observe a Mg^2+^-dependent intensity change in Trp fluorescence for zGCAP1,2,3,4 and 5, although we observed in the gel shift assay that Mg^2+^ has an influence on the electrophoretic mobility indicating binding of Mg^2+^ to zGCAPs. Among all zGCAPs, zGCAP7 has a unique distribution of Trps in the amino acid sequence, present at positions 94, 164 and 178. Although a Trp is missing at position 51, we observed for zGCAP7 a Mg^2+^-effect below 1 µM free [Ca^2+^] (Table 1 and Figure 4). Therefore, we conclude that position 164 and/or 178 might compensate for lacking Trp in position 51 and is/are involved in changes of Trp fluorescence emission below 1 µM free [Ca^2+^].

In summary, our data indicate the existence of a complex interplay between Ca^2+^ and zGCAPs, of which each zGCAP form displayed a very specific detection mode for Ca^2+^. In photoreceptor cells cytoplasmic [Ca^2+^] is expected to be balanced at levels that finally depend on the illumination conditions [46]. The six different zGCAPs could operate in cones according to the Ca^2+^-relay model previously hypothesized for the function of GCAP1 and GCAP2 in bovine rods [22]. The Ca^2+^-fluctuations in a cone cell are therefore adjusted by the Ca^2+^-homeostasis that is under control of the illumination conditions. Each range of cytoplasmic [Ca^2+^] would be optimal for a specific zGCAP to operate as an activator of GC. The differential fingerprint properties of zGCAPs fit into a step by step regulation of GC activity triggered by changing [Ca^2+^]. This zGCAP signaling system might be part of the molecular toolbox enabling the cones to respond to a large range of light intensities. The specific challenges of the zebrafish habitat, for instance, the dominance of short wavelengths in ambient background light, probably caused genome duplication under evolutionary pressure, which is now mirrored in the diversity of zGCAP expression (for an extensive discussion on this point see [47].
